# The Genetic Evidence of Burn-Induced Cardiac Mitochondrial Metabolism Dysfunction

**DOI:** 10.3390/biomedicines8120566

**Published:** 2020-12-03

**Authors:** Jake J. Wen, Claire B. Cummins, Taylor P. Williams, Ravi S. Radhakrishnan

**Affiliations:** Department of Surgery, University of Texas Medical Branch, Galveston, TX 77555, USA; cbcummin@utmb.edu (C.B.C.); tpwillia@utmb.edu (T.P.W.)

**Keywords:** burn injury, cardiac dysfunction, gene profiling, mitochondrial metabolism, oxygen consumption

## Abstract

Burn-induced cardiac dysfunction is thought to involve mitochondrial dysfunction, although the mechanisms responsible are unclear. In this study, we used our established model of in vivo burn injury to understand the genetic evidence of burn-induced mitochondrial confusion dysfunction by describing cardiac mitochondrial metabolism-related gene expression after burn. Cardiac tissue was collected at 24 hours after burn injury. An O2K respirometer system was utilized to measure the cardiac mitochondrial function. Oxidative phosphorylation complex activities were determined using enzyme activity assays. RT Profiler PCR array was used to identify the differential regulation of genes involved in mitochondrial biogenesis and metabolism. The quantitative qPCR and Western blotting were applied to validate the differentially expressed genes. Burn-induced cardiac mitochondrial dysfunction was supported by the finding of decreased state 3 respiration, decreased mitochondrial electron transport chain activity in complex I, III, IV, and V, and decreased mitochondrial DNA-encoded gene expression as well as decreased levels of the corresponding proteins after burn injury. Eighty-four mitochondrial metabolism-related gene profiles were measured. The mitochondrial gene profile showed that 29 genes related to mitochondrial energy and metabolism was differentially expressed. Of these 29 genes, 16 were more than 2-fold upregulated and 13 were more than 2-fold downregulated. All genes were validated using qPCR and partial genes were correlated with their protein levels. This study provides preliminary evidence that a large percentage of mitochondrial metabolism-related genes in cardiomyocytes were significantly affected by burn injury.

## 1. Introduction

Severe burns have a significant impact on several organ systems. Cardiovascular dysfunction following a severe burn was first described in 1961 [1]. Burn-induced cardiac dysfunction manifests as tachycardia, systolic heart failure, and increased energy expenditure [1]. Half of all pediatric patients with severe burns demonstrated systolic dysfunction, which is associated with longer hospital stays and an increase in the number of surgical interventions [2]. Cardiac dysfunction continues past the acute phase of injury, with elevated energy expenditure, tachycardia, and diminished cardiac output sometimes lasting for more than a year following the initial injury [3,4]. Common hypotheses for cardiac dysfunction after burn include excessive beta-adrenergic stimulation, cytokine stimulation, nitric oxide production, and abnormalities in calcium homeostasis leading to cardiomyocyte apoptosis [5]; however, the specific mechanism has not yet been characterized in detail.

In other organ systems, burns induce mitochondrial dysfunction. Burns acutely increase the respiratory capacity and function of liver mitochondria [6]. Skeletal muscle is particularly sensitive to mitochondrial damage after burn [7,8,9]. Mitochondrial-targeted antioxidants have been used to promote the recovery of skeletal muscle function following a burn [10]. Mitochondrial dysfunction has been linked to cardiac dysfunction in the setting of heart failure, cardiac arrhythmia, and diabetic heart disease [11,12,13,14]. Mitochondrial dysfunction in cardiomyocytes after a burn injury has been identified [15], but the underlying mechanisms remain unclear.

Polymerase chain reaction (PCR)-based array and gene profiling have been used to better understand mitochondrial dysfunction in a variety of other disease processes. Mitochondria are not only the energy generator organelles, but also play a role in apoptosis, proliferation, and redox homeostasis. In prostate cells, the PCR array was able to identify mitochondrial genes linked to the malignant transformation and disease progression [16]. Similarly, the differential expression of mitochondrial genes was able to predict ulcerative colitis-associated colorectal adenocarcinoma [17]. Microarray analysis has been used to detect genes which are affected by severe burns at different time points, leading to a deeper understanding of the molecular mechanisms surrounding burns [18]. Pathway-focusing qPCR-based arrays would be accurate, time saving and targeted.

In this study, we confirmed that burn-induced cardiac dysfunction was associated with changes in mitochondrial respiration, and we used PCR arrays to identify the differential expression of genes involved in mitochondrial biogenesis and metabolism in cardiomyocytes after burn.

## 2. Experimental Section

### 2.1. Ethics Statement

All animal work followed the experimental animal use protocol under the National Institutes of Health guidelines and was certified by our Institutional Animal Care and Use Committee (IACUC, Protocol number: 1509059, Approval Date: August, 2018).

### 2.2. Animal Model

Male Sprague-Dawley rats (300–350 g, Harlan Laboratories, Indianapolis, IN, USA) could adapt in animal housing at 25 °C and on a 12:12 light–dark cycle for at least one week prior to the experimentation. A well-established model for 60% total body surface body area (TBSA) full-thickness scald burn was utilized as we have previously published [1,2,3]. In detail, the rats were injected with buprenorphine (0.05 mg/kg s.c.) for analgesia and anesthetized with isoflurane (3–5%). The rats were placed within a self-made protective frame mold that exposed ~30% TBSA and submerged on both the dorsal (10 s) and ventral sides (2 s) in 95–100 °C water, resulting in a 60% TBSA burn in total. Lactated ringer (LR) solution (40 mL/kg body weight, i.p.) was administered immediately after the burn for resuscitation, and the rats received oxygen during the recovery from anesthesia. Analgesia (buprenorphine, 0.05 mg/kg) was given as needed every 6 h after the burn due to damage to nerve endings in full-thickness burns. For sham control, the animals underwent all the same procedures as those in the burn group, except for submersion in room temperature water instead of 95–100 °C water. At 24 h post burn (hpb), rats were humanely euthanized by bilateral thoracotomy under anesthesia (≥5% isoflurane) to collect left ventricle tissue. Heart tissue for PCR array and qPCR work was collected in RNAprotect Tissue Reagent (Qiagen, Germantown, MD, USA, Cat# 76104). Heart tissue for mitochondrial oxygen consumption was placed in a biopsy preservation solution (BIOPS) buffer (10 mM CaK2-EGTA, 7.2 mM K2-EGTA, 20 mM taurine, 50 mM K-MES, 0.5 mM dithiothreitol, 6.5 mM MgCl_2_, 5.8 mM ATP, and 15 mM creatine phosphate; pH 7.1). Heart tissue for oxidative phosphorylation complex activities were collected in Eppendorf tubes and started at −80 °C.

### 2.3. Preparation of Permeabilized Fibers from Trout Heart

Permeabilized fibers from rat heart muscle were prepared according to the protocol of Pesta and Gnaiger (2012) [19]. Briefly, a sacrificed heart was placed in a falcon containing BIOPS buffer (10 mM CaK2–EGTA, 7.2 mM K2 EGTA, 20 mM imidazole, 20 mM taurine, 50 mM K-MES, 0.5 mM dithiothreitol, 6.5 mM MgCl_2_, 5.8 mM ATP, and 15 mM creatine phosphate; pH 7.1) and the connective tissue and capillaries were removed. Myocardial tissue samples were taken from the inner wall of the left ventricle and fiber bundles were separated mechanically with two pairs of very sharp angular forceps. Proper separation was observed within 1–2 min of treatment, visualized by a change in color from red to pale. Fiber bundles were quickly transferred into 2 mL BIOPS containing freshly prepared saponin (50 µg/mL BIOPS) and incubated on a shaker on ice for 30 min, then transferred into 2 mL of MIR05 buffer (0.5 mM EGTA, 3 mM MgCl_2_, 60 mM K-lactobionate, 20 mM taurine, 10 mM KH_2_PO_4_, 20 mM HEPES, 110 mM sucrose, and 1 mg/mL fatty acid-free BSA, pH 7.1) and then gentle agitation was continued for 10 min on ice. After permeabilization, approximately 2 mg/chamber was determined and transferred into the medium equilibrated at 15 °C in the O2k-chamber to run oxygen consumption.

### 2.4. O2K Respirometer System

Approximately 2 mg of myofiber bundles were used for measuring mitochondrial respiration with an O2K respirometer (Oroboros Instruments, Innsbruck, Austria). Oxygen concentration was determined at 2 s intervals and analyzed by using Oroboros DatLab 7.4 software. In brief, a leak respiratory state was recorded with myofiber bundles alone, and state 4 respiration supported by electron flow through complex I (5 mM pyruvate, 10 mM glutamate, and 2 mM malate, P+G+M) was recorded. Electron transfer was coupled to phosphorylation by the addition of 5 mM ADP, and state 3 respiration supported by complex I was recorded. Maximal state 3 respiration with parallel electron input from complex I and complex II was recorded with an addition of 10 mM succinate, and complex II supported respiration was measured in the presence of 6.25 µM rotenone. Cytochrome C (10 µM) was added to assess the competence of the outer mitochondrial membrane. The absence of a significant increase in respiratory flux after the addition of cytochrome C indicates that the outer mitochondrial membranes are intact. Finally, maximal electron transfer capacity was recorded in the presence of 5 µM Carbonyl cyanide p-(trifluoro-methoxy) phenyl-hydrazone (FCCP).

### 2.5. Isolation of Cardiac Mitochondria

Cardiac mitochondria were isolated and purified using the MitoCheck^®^ Mitochondrial (Tissue) Isolation Kit (Cayman, Ann Arbor, MI, USA, Cat# 701010) following the manufacturer’s instructions. Briefly, the fresh heart tissue was washed, suspended in mitochondrial homogenization buffer containing protease inhibitor (Abcam Cat# ab201111) to be homogenized, and spun at 680× *g* for 15 min. The supernatant was transferred to a new tube and then centrifuged again at 8800× *g* for 15 min. Mitochondrial pellets were washed twice using mitochondrial homogenization buffer. Mitochondrial protein concentration was determined by bicinchoninic acid assay (Pierce™ BCA Protein Assay Kit, ThermoFisher, Waltham, MA, USA, Cat# 23225). Aliquoted mitochondrial pellets were stored at −80 °C for further usage.

### 2.6. Oxidative Phosphorylation Complex Activities

Oxidative phosphorylation complex activities were measured using MitoCheck Complex Activity Assay Kits (Cayman Chemical, Ann Arbor, MI, USA, Cat# 700930 for complex I, Cat# 700940 for complex II, Cat# 700950 for complex III, Cat# 700990 for complex IV, and Cat# 701000 for complex V) according to the manufacturer’s protocols. Briefly, complex I activity was determined by measuring the decreased rate of NADH oxidation at 340 nm; complex II activity was determined as a decrease in absorbance at 600 nm over time; complex III activity was determined by measuring the reduction of cytochrome c at 550 nm; complex IV activity was determined by the oxidation rate of reduced cytochrome c at 550 nm; and complex V activity was determined by the rate of NADH oxidation at 340 nm. Results were presented as a percent of activity, based on the manufacturer’s protocols, following the format: complex activity (%) = (rate of sample wells/rate of control] × 100.

### 2.7. Mitochondrial Copy Number

Qiagen DNeasy Blood & Tissue Kit (Qiagen, Germantown, MD, USA, Cat# 69504) was applied to extract genomic DNA following the manufacturer’s instruction. To estimate mitochondrial DNA (mtDNA) copy number, the rat control-region (D-loop) and nuclear GAPDH were amplified by qPCR using specific primers (D-Loop forward: CGGATGCCTTCCTCAACATA and reverse: AGTCTTTCGAGCTTTGTCTATGA. KF011917.1; GAPDH forward: ACTCCCATTCTTCCACCTTTG and reverse: CCCTGTTGCTGTAGCCATATT. NM_017008.4). The ratio of the mitochondrial copy number was presented as a copy number of the mitochondrial genome to the copy number of the nuclear housekeeping gene (GAPDH).

### 2.8. RNA Isolation

Ten milligrams of cardiac tissue were utilized to isolate and purify the total RNAs using RNeasy Mini Kit (Qiagen, Gaithersburg, MD, USA, Cat# 74104) according to the manufacturer’s instruction. The purified RNAs were treated with DNAse I and RNAse-free (NEB, Westlake, LA, USA, Cat# M0303S) to digest contaminated genomic DNA. To judge the integrity and overall quality of the isolated RNA, 2 µg of RNA were run on 1% native agarose gels. Spectrophotometer (The DU^®^ 700 UV/Visible, Beckman Coulter, Pasadena, CA, USA) was applied to measure the RNA quantitatively by the determination of absorbance at 260 and 280 nm (OD260/280 ratio ≥ 2, 1 OD260 Unit = 40 µg/mL RNA).

### 2.9. First-Strand cDNA Synthesis

The cDNA synthesis mixture (13 μL) containing 2 μg of total RNA, 1 μL of 500 ng oligo(dT)18, 1 μL of 10 mM dNTP mix and sterile RNase-free water was heated to 65 °C for 5 min to denature the RNA and incubate on ice for 1 minute. To the reaction tube was added 4 μL 5× first-strand buffer, 1 μL 0.1 M DTT, 1 μL RNase inhibitor, and 1 μL SuperScript III reverse transcriptase after centrifugation, which was then mixed by pipetting gently and incubated at 42 °C for 60 min. The reaction was inactivated by heating at 95 °C for 15 min and diluted to a final volume of 102 μL with ddH_2_O and stored at −80 °C.

### 2.10. Real-Time PCR Array and qPCR

RT^2^ Profiler^TM^ PCR Array Rat Mitochondrial Energy Metabolism (Qiagen, Gaithersburg, MD, USA, PARN-008Z) was used to identify and pool the differentially expressed gene profiles that were related to mitochondrial biogenesis and bioenergetics function. The Rat profile PCR array profiles the expression of 84 key genes associated with mitochondrial respiration including complex I, II, II, IV, and V genes as well as some pathway activity signature genes. In brief, a reaction mixture was prepared by successively adding 550 μL of 2× RT^2^ SYBR^®^ Green qPCR Mastermix, 102 μL of the diluted first-strand cDNA synthesis reaction and 448 μL of ddH_2_O. The mixture was then administrated to the PCR array and performed PCR on a Bio-Rad^®^ iCycler under 95 °C for 10 min, 40 cycles of 95 °C for 15 s, and 60 °C for 1 min. The results were analyzed by the ΔΔCt method. For qPCR, we designed the primers (Table 1, Table 2, Table 3 and Table 4) based on the identified differentially expressed genes using NCBI Primer-BLAST and the PCR running system was the same as PCR array.

### 2.11. Quantitative Western Blotting (WB)

Approximately 100 mg of frozen heart tissue samples were lysed for 20 min on ice in 500 μL radioimmunoprecipitation assay (RIPA) lysis buffer (Abcam, Cambridge, MA, USA, Cat# ab156034) containing one protease inhibitor cocktail tablet (Roche, Indianapolis, IN, USA, Cat# 11697498001) per 10 mL lysis buffer, and homogenized by using homogenizer. Cellular debris was removed by centrifugation at 4 °C. The protein concentration was determined as 2.4, and 20 μg protein were separated by standard 12% SDS-PAGE gels. The separated proteins were then transferred on to PVDF membranes. The membranes were then sequentially blocked for 1 h in LiCor PBS blocking buffer and incubated with primary and secondary antibodies for 1 h, separately, with 4 × 5 min PBS washes in between. Primary antibodies used were at 1:1000 dilution and included rabbit polyclonal anti-COX17 antibody (Abcam; #ab69611), rabbit monoclonal anti-NDUFS4 antibody (Abcam; #137064), rabbit monoclonal anti-UQCRFS1 antibody (Abcam, #191079), rabbit polyclonal anti-UCP1 antibody (Cell Signaling, #14670), rabbit monoclonal anti-ND1 antibody (Abcam, #ab181848), rabbit polyclonal anti-CYTB antibody (Antibodies-online, #ABIN2773962), mouse monoclonal anti-COX II antibody (Abcam, #ab110258), rabbit polyclonal anti-ATP6 antibody (Abcam, #ab175299), rabbit polyclonal anti-beta actin antibody (Abcam, #ab8227) and mouse monoclonal anti-GAPDH (Cell Signaling, #51332). Secondary antibodies used at 1:5000 dilution were IRDye 680 goat antirabbit IgG (Licor, #926-68071) and IRDye 800 goat antimouse IgG (Licor, 926-32210). Membranes were scanned and bands were quantified with the Licor Odyssey CLx. This system allows the accurate quantification of proteins on Western blotting (WB) across a wide linear range and probing the same membrane with different antibodies raised in different species (e.g., mouse and rabbit) at the same time.

### 2.12. Statistical Analysis

All experiments were carried on triplicate observations per sample (*n* = 6–8 mice/group). All data were presented as the mean ± standard error mean (SEM) and analyzed using GraphPad Prism8.2 software. Briefly, the Kolmogorov–Smirnov test under Column Statistics was applied to determine if the data were normally distributed or not. If normally distributed, the data were analyzed by Student’s t test (comparison of two groups). If not normally distributed, the Mann–Whitney (comparison of two groups) tests were performed. Significance is expressed by *24 hpb rats vs. sham rats) (* *p* < 0.05, ** *p* < 0.01, *** *p* < 0.001). Regarding PCR array data, the data analysis was conducted using the ΔΔCt module at the Qiagen Gene Globe Data Analysis Center portal. The efficiency of all the primers used in the kits has been shown to be over 90%. The RT-PCR arrays contain control wells/samples for the determination and/or verification of rat genomic contamination, reverse transcription control, and positive PCR controls. At least five reference genes, including beta-actin (ACTB), glyceraldehyde-3-phosphate dehydrogenase (GAPDH), beta-2-microglobulin (B2M), hypoxanthine phosphoribosyl transferase 1 (HPRT1), and ribosomal protein, large, P0 (RPLP0), were used for the data normalization. In these analyses, genes with a greater than 2-fold change in expression at a value of *p* < 0.05 between burn and sham group rats were defined as differentially expressed and selected for inclusion in comparative analyses.

## 3. Results

### 3.1. Burn Induces Cardiac Mitochondrial Dysfunction

**Figure 1:**
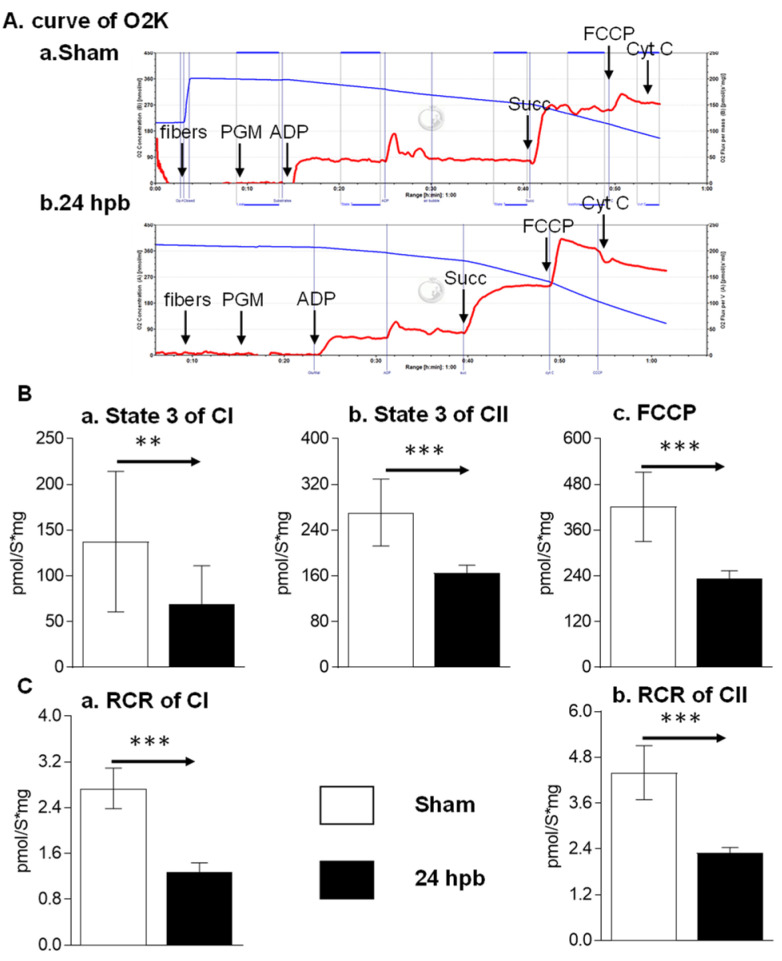
Cardiac mitochondrial respiration in the sham (*n* = 9) and burn groups (*n* = 9): (**A**) original mitochondrial respiration curve, PGM: pyruvate, glutamate and malate; ADP, Adenosine diphosphate; Succ: succinate; FCCP, protonophore carbonyl cyanide p-trifluoromethoxy-phenylhydrazone; Cyt C, cytochrome c. Oxygen concentration ([μM] blue line) and oxygen flux per tissue mass (pmol·s^−1^·mg^−1^ wet weight) (red line) are displayed. Black arrows showed when the substrates/reagents were injected. (**B**) electron flow was supported with complex I (**a**), complex II (**b**), and the uncoupling agent (**c**); (**C**) respiratory control ratios (RCRs) energized by complex I substrate (**a**) and complex II substrate (**b**) were calculated as the ratios between state 3 and state 4 rates. Data are triplicated (*n* = 9) and plotted as the mean ± SD. Significance is denoted as follows: ** (*p* < 0.01), *** (*p* < 0.001).

To determine if a burn would induce cardiac dysfunction via interference with cardiac mitochondrial energy production, we used the O2K respiration system. The original respiration curves were shown in Figure 1A. Burn injury decreases state 3 respiration supported by complex I activity by 49.8% (Figure 1Ba) and state 3 respiration supported by complex II activity by 39.2% (Figure 1Bb). The burn also decreased FCCP-induced uncoupled ATP generation capacity by 44.39% (Figure 1C). The respiratory control ratio (RCR) is a surrogate of mitochondrial coupling state. The burn induced a significant decrease in the RCR of both complex I and complex II substrate energized oxygen consumption (Figure 1B). This indicates that burn injury induces cardiac mitochondrial dysfunction.

### 3.2. Burn Induces Cardiac Mitochondrial Electron Transport Chain Dysfunction

**Figure 2:**
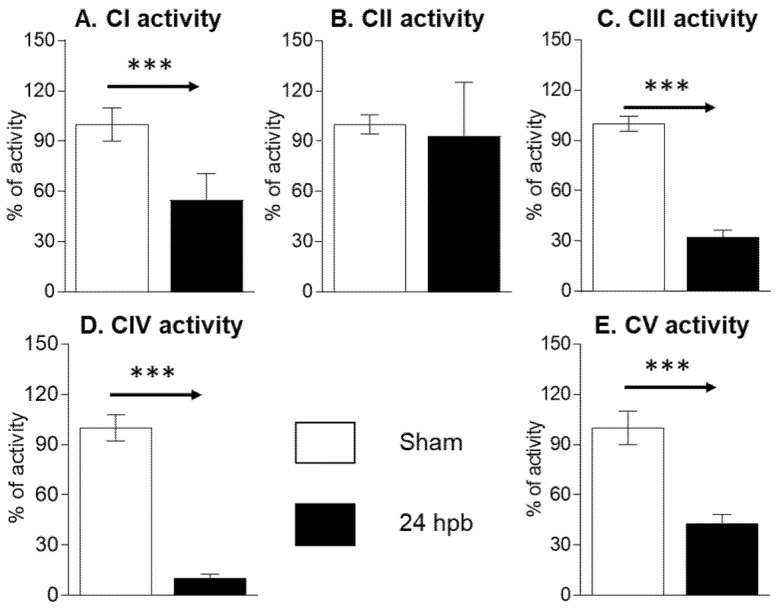
Cardiac mitochondrial electron transport chain activity in sham (*n* = 9) and burn groups (*n* = 9): (**A**) complex I; (**B**) complex II; (**C**) complex III; (**D**) complex IV; and (**E**) complex V are shown. Data are triplicated (*n* = 9) and plotted as the mean ± SD. Significance is denoted as follows: *** (*p* < 0.001).

The MitoCheck Complex Activity Assay Kits were used to measure complex I–V activity. Burn injury led to a 45.07% decrease in complex I activity (Figure 2A), no significant change in complex II activity (Figure 2B), a 67.16% decrease in complex III activity (Figure 2C), an 89.3% decrease in complex IV activity (Figure 2D), and a 56.63% decrease in complex V activity (Figure 2E). This suggests that mitochondrial dysfunction is associated with mitochondrial complex activity dysfunction.

### 3.3. Analysis of Burn-Induced Cardiac Mitochondrial Metabolism-Related Gene Expression

**Figure 3:**
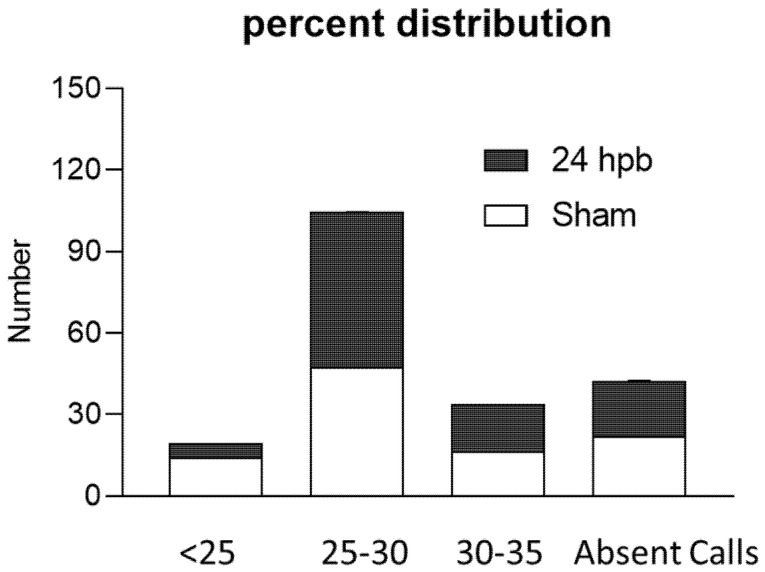
Percent distribution of C_T_ values in the sham (none filling column) and burn groups (black column).

The RT^2^ Profiler^TM^ PCR Array was used to study burn-induced changes in cardiac mitochondrial metabolism-related gene expression. All the data were normalized using five housekeeper genes. Based on the cycle threshold (C_T_) the genes expressed in the sham and burn groups were clustered into five categories including <25, 25–30, 30–35, 35–40, and not detectable (Figure 3). Burn injury significantly changed the distribution of genes across these categories by decreasing the <25 group from 14.24% to 5.21% and increasing the 25–30 group from 47.57% to 59.94% (Figure 3).

**Figure 4:**
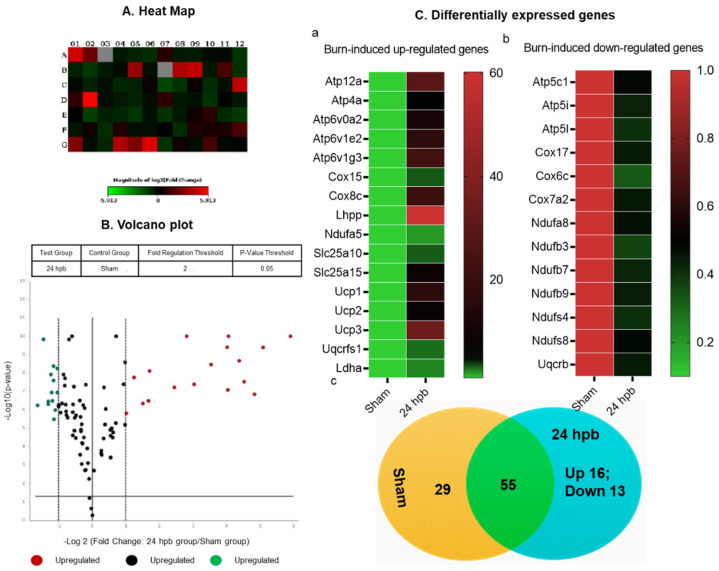
The analysis of burn-induced cardiac metabolism-related gene expression; (**A**) original heat map demonstrates upregulated genes in red and downregulated genes in green; (**B**) volcano plot demonstrates significant gene expression vs. fold change on the y and x axes, respectively. Red represents the upregulated genes, green represents downregulated genes and black represents no significant change; (**C**) shows upregulated genes (**a**), downregulated genes (**b**), and summary (**c**) after burn.

After the analysis utilizing the ΔΔC_T_ method, the results were depicted in Figure 4. The heat map demonstrates gene expression by color density; red indicates gene upregulation and green indicates gene downregulation (Figure 4A). Similarly, a volcano plot revealed differentially expressed genes based on fold-change (Figure 4B). Fifteen genes were over expressed in the burn compared to the sham group (Figure 4Ca) and thirteen genes were under expressed (Figure 4Cb). These genes are listed in Figure 4C. Finally, a cluster gram of differentially expressed genes is presented in Figure 4C.

### 3.4. Burn-Induced Downregulated Cardiac Mitochondrial Metabolism-Related Gene Expression

**Figure 5:**
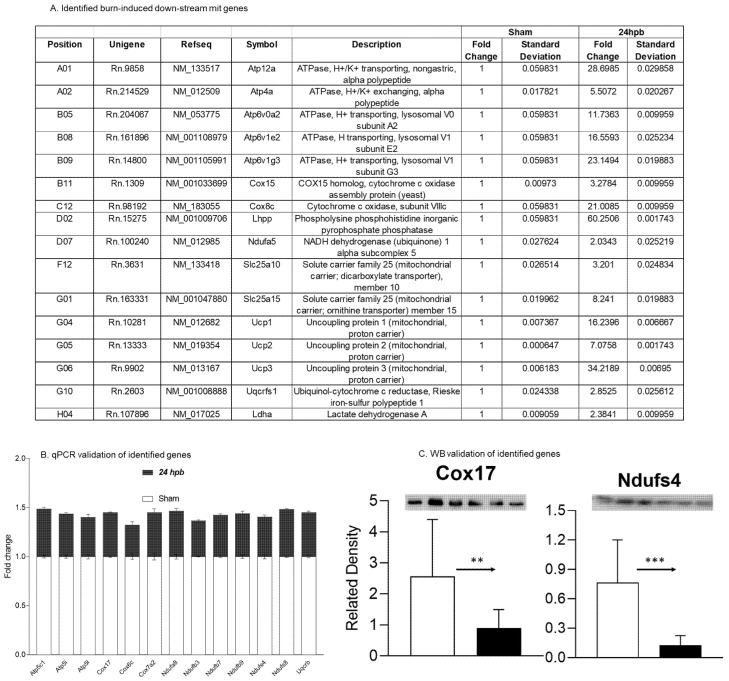
Downregulated cardiac metabolism-related genes after burn: (**A**) names, locations, fold change, *p*-values, and NCBI gene IDs of identified genes; (**B**) data validation of identified downregulated genes with qPCR; and (**C**) data validation of identified downregulated genes with Western blotting (WB). ** *p* < 0.01 and *** *p* < 0.001 (Sham vs. 24 hpb).

Thirteen differentially expressed genes were downregulated in the cardiac mitochondria after burn (Figure 5A). Six of the thirteen genes were associated with cardiac mitochondrial complex I, including Ndufa8, Ndufb3, Ndufb7, Ndufb9, Ndufs4, and Ndufs8 (Figure 5A). One gene was associated with complex III (Uqcrb), three genes were related to complex IV (Cox 17, Cox6c, and Cox7a2) and three genes were associated with complex V (Atp5C1, Atp5i, and Atp5l). To validate these findings, qPCR was utilized in cardiac cDNA pools and the results are shown in Figure 5B. To evaluate if burn-induced downregulated genes would interfere with their encoded protein production, the protein levels in heart tissues of Cox17 and Ndufs4 were measured by WB (Figure 5C) and were found to be significantly decreased by 65% and 84%, respectively.

### 3.5. Burn-Induced Upregulated Cardiac Mitochondrial Metabolism-Related Gene Expression

**Figure 6:**
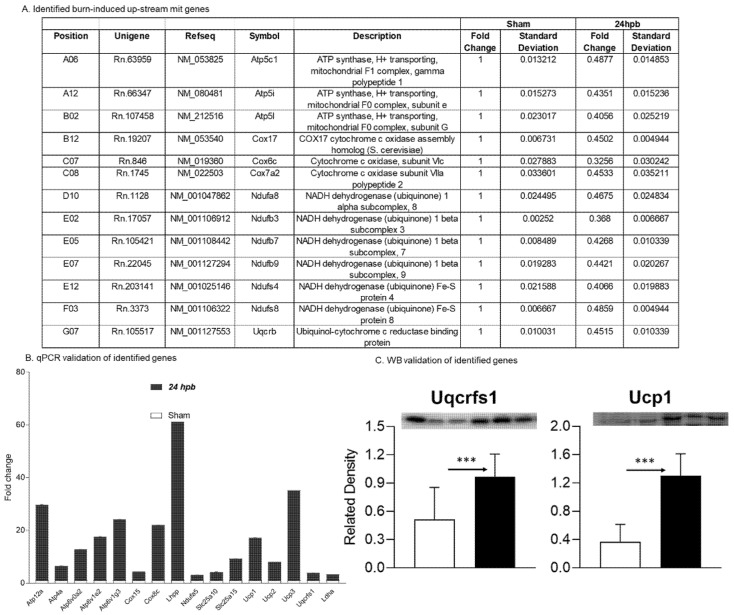
Upregulated cardiac metabolism-related genes after burn: (**A**) names, locations, fold change, *p*-values, and NCBI gene IDs of identified genes; (**B**) data validation of identified downregulated genes with qPCR and Western blotting (**C**). *** *p* < 0.001 (Sham vs. 24 hpb).

Sixteen differentially expressed genes were upregulated in cardiac mitochondria after burn (Figure 6A). One of the sixteen genes was associated with cardiac mitochondrial complex I (Ndufa5), one was associated with complex III (Uqcrfs1), two genes were associated with complex VI (Cox 15 and Cox8c), six genes were associated with complex V (Atp12a, Atp4a, Atp6v0a2, Atp6v1g3, and Lhpp), five genes were associated with accessory proteins (Slc25a10, Slc25a15, Ucp1, Ucp2, and Ucp3) (Figure 6A), and one was related to pyruvate metabolism (Ldha). To validate these findings, qPCR was utilized in cardiac cDNA pools and the results are shown in Figure 6B. To evaluate if burn-induced upregulated genes would correlate with their encoded protein production, the protein levels in heart tissues of Uqcrfs1 and Ucp1 were measured by WB (Figure 6C) and were found to be significantly increased by 88.4% and 2.54-fold, respectively.

### 3.6. Downregulation of Cardiac Mitochondrial DNA-Encoded Genes and Proteins after Burn

**Figure 7:**
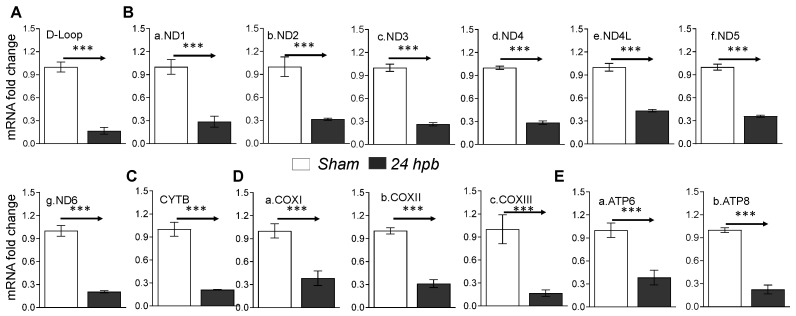
mtDNA replication and mtDNA-encoded gene expression in burned rats. Sprague Dawley rats were burned with boiling water and treated. Heart tissues were collected at 24 h post burn (24 dpb) to make the heart gDNAs, RNAs and cDNAs. qPCR was utilized to measure the mtDNA copy number and mtDNA-encoded 13 gene expressions. (**A**) the myocardial levels of the mt D-Loop copy number are shown; (**B**) panels a–g show representatives of gene expressions for the mtDNA-encoded complex I genes including ND1, ND2, ND3, ND4, ND4L, ND5 and ND6; (**C**) the mtDNA-encoded complex III gene, CYTB, is shown; (**D**) panels a–c show the mtDNA-encoded complex IV genes including COXI, COXII and COXIII; and (**E**) panels a,b show the gene expressions of mtDNA-encoded complex V genes including ATP6 and ATP8. In all figures, the data are presented as the mean value ± SD. Significance is shown as and presented as *** *p* < 0.001 (24 hpb vs. matched control, *n* = ≥6 per group).

**Figure 8:**
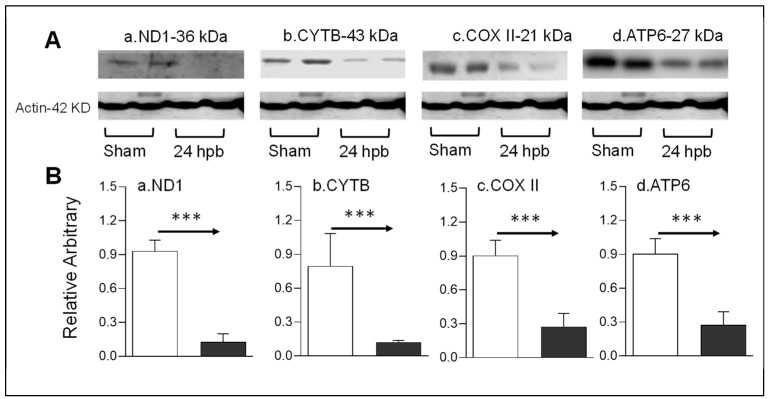
mtDNA-encoded proteins in burn injury myocardium. Rats were burned with boiling water and harvested at 24 h post burn: (**A**) Western blotting—heart tissue homogenates were subjected to Western blotting with anti-ND1antibody (**Aa**), anti-CYTB antibody (**Ab**), anti-COX II antibody (**Ac**) and anti-ATP6 antibody; (**B**) densitometry analysis of the panel A signals (**Ba**–**Bd**) is presented. Densitometry analysis of the WB bands in the heart homogenates were normalized to β-actin. Data are plotted as the mean value ± SEM (*n* = 6–10 rats per group). Significance is shown as *** *p* < 0.001.

Mitochondrial energy/metabolism gene profiles demonstrated that parts of nuclear-encoded mitochondrial metabolism-related genes were upregulated after the burn, suggesting that not all nuclear-encoded oxidative phosphorylation-related genes have the same function in mitochondrial oxidative phosphorylation and are a compensatory-like mechanism only at 24 h post burn. We also have another hypothesis: that mitDNA-encoded genes may play a more important role in burn injury. qPCR was applied to measure 13 mtDNA-encoded genes and found that all 13 genes were significantly decreased by 56% to 83% in complex I, 79% in complex III, 62% to 83% in complex IV and 62% to 77% in complex V, respectively, after burn (Figure 7). To know if the alteration of mtDNA-encoded genes were in correlation with the corresponding translated proteins, one representative protein of each complex including ND1 (complex I), Cyt B (complex III), Cox II (complex IV) and ATP6 (complex V) was subjected to Western blotting. The selected mtDNA-encoded protein levels were decreased by 86.4% for ND1 (Figure 8Aa,Ba), 84.5% for Cyt B (Figure 8Ab,Bb), 69.5% for COX II (Figure 8Ac,Bc) and 69.9% for ATP6 (Figure 8Ad,Bd) after the burn.

## 4. Discussion

In this study, we demonstrated that burn injuries caused cardiac mitochondrial dysfunction based on changes in cardiac mitochondrial oxygen consumption and electron transport chain activities. Then, we used PCR profile arrays to identify the differentially expressed genes associated with mitochondrial metabolism. Thirteen mitochondrial proteins were downregulated after burn injury and fifteen genes were upregulated, which may be correlated with their encoded protein levels. To our knowledge, this is the first study to explore burn-induced cardiac metabolism-related gene expression. These data will provide a foundation for future research by furthering the understanding of burn-induced cardiac dysfunction.

Few publications have explored the relationship between burn-induced cardiac and mitochondrial dysfunction. Liang et al. reported that cardiac mitochondrial function was decreased after burn injury by determining the activity of mtNOS, mtPLA, the F(0)F(1)-ATPase synthetic activity, and the activation of cytochrome c oxidase, and mitochondrial calcium levels [20]. Similarly, Zang et al. studied the cardiac mitochondrial response to burns by measuring lipid peroxidation, mitochondrial outer membrane damage, cytochrome c translocation, and the activities of SOD and glutathione peroxidase [15]. Our findings of significantly decreased cardiac mitochondrial oxygen consumption and electron transport chain activities strongly support these previous publications. However, our study is the first to directly quantify the difference in mitochondrial function after burning instead of using adjuncts for mitochondrial function.

The advantage of using RT² Profiler PCR Arrays is to enable the quick, reliable gene expression analysis of specific targeted pathways. The arrays are pathway-focused panels of laboratory-verified qPCR assays, with integrated and patented controls to provide high reliability. The arrays have high sensitivity, with as little as 1 ng or as much as 5 µg of total RNA per array plate needed to provide greater than 80% present call rates. The arrays have high reproducibility, with strong correlations across technical replicates, lots, and instruments; the arrays have high specificity with high-quality input RNA and yield single bands of the predicted size without primer-dimers or other secondary products. Therefore, PCR arrays provide highly accurate real-time PCR results. In addition, PCR arrays have multiples references to normalize data and avoid alterations in traditional housekeeping genes that occur in different conditions. This study strongly supports the merits of PCR arrays.

The Rat Mitochondrial Energy Metabolism RT² Profiler PCR Array profiles the expression of 84 key genes involved in mitochondrial respiration, including the genes encoding components of the electron transport chain and oxidative phosphorylation complexes. Five genes, all related to accessory proteins, were found to be significantly changed after burn. Principal among these were Ucp1, Ucp2, and Ucp3, all of which were upregulated. Uncoupling proteins (UCPs) perturb respiratory coupling by inducing a proton leak through the mitochondrial membrane [21]. Ucp1, or thermogenin, dissipates the proton gradient in an energy-wasting manner, generating heat instead of ATP [22]. Ucp2 and Ucp3 also dissipate the mitochondrial electrical potential, but do so in order to reduce the production of the superoxide radicals at complex I in the setting of ischemia [23]. Heart failure results in a decrease in Ucp2 and Ucp3, though it is unknown whether the change in these proteins is beneficial or detrimental to the failing heart [21]; however, an increase in the expression of UCPs in cardiac mitochondria decreases free radical production. This plays a key role in the development and progression of heart failure [24,25]. Interestingly, these genes were upregulated after burn, indicating that they could potentially be cardio-protective in the development of burn-induced cardiac dysfunction.

Six complex I-associated genes were downregulated after burn. All of these genes code for a subunit of complex I which has NADH dehydrogenase and oxidoreductase activity. Complex I contributes to most of the ROS generated in intact mitochondria, which are subsequently directly or indirectly involved in further signaling pathways, such as apoptosis [26,27]. Ndufs4, a peripheral membrane protein located on the matrix side of the inner mitochondrial membrane [28,29], is one of the genes significantly downregulated after a burn injury. The deficiency of Ndufs4 is associated with hypertrophic cardiomyopathy, as well as leukoencephalopathy and lethal infantile mitochondrial disease [30]. Interestingly, Ndufs8 and Ndufa5, are components of complex I, which we found to be upregulated after a burn injury. Ndufs8, like Ndufs4, is a peripheral membrane protein which can contribute to hypertrophic cardiomyopathy [31]. Given these changes, it is likely that burn-induced cardiac mitochondrial dysfunction is potentially affected by alterations in complex I-related gene expression.

Two complex III-associated genes were significantly altered in burns: Uqcrb and Uqcrfs1. Uqcrb plays an important role in electron transfer as part of complex III and helps with complex III maintenance [32,33,34]. Uqcrb plays an role in angiogenesis, and represents a target for anti-angiogenic activity [32,33,34]. Uqcrfs1 is the cleavage product of the Rieske protein and is the penultimate step in complex III assembly. Surprisingly, Uqcrb was downregulated after burn while Uqcrfs1 was upregulated. Future research could help delineate the role of complex III in burn-induced cardiac dysfunction.

Five complex IV-associated genes were significantly changed after burn injury. Complex IV, also known as cytochrome c oxidase (COX), catalyzes the transfer of electrons from reduced cytochrome c to the final acceptor of electrons, O_2_ [35]. COX17, one of the downregulated complex IV genes, is essential for the assembly of functional cytochrome c oxidase, the cristae organizing system complex, and for the delivery of copper ions to the mitochondria for insertion into the enzyme [36]. Mutations of COX17 have been linked to respiratory defects [37]. COX15, one of the upregulated complex IV genes, is a key enzyme involved in heme-a biosynthesis and the over-expression of COX15 has been linked to the development of Alzheimer’s disease [38]. Similar to complex III, how the differential expression of these genes after burning contributes to the decrease in complex IV activity has not yet been fully elucidated.

Finally, the expression of nine complex V-associated genes were significantly altered after burn. Complex V is also known as human mitochondrial ATP synthase, and synthesizes ATP from ADP in the mitochondrial matrix using the energy provided by the proton electrochemical gradient [39,40,41]. Atp12a, one of the upregulated genes, is of particular note due to its association with cardiac disease. Atp12a is the no gastric form of the H^+^/K^+^-ATPase [42,43]. Left ventricular diastolic function is associated with a genetic variation in the Atp12a promotor, resulting in superior myocardial relaxation as compared to non-carriers [44]. Additionally, Kinoshita et al. demonstrated that lower Atp12a expression might contribute to the development of idiopathic hypertension [45]. While an overall decrease was seen in the functionality of complex V after burn, the upregulation of Atp12a may be a protective mechanism for cardiac function following injury.

Future research is still required to understand the effects of cardiac mitochondrial damage in burn-induced heart dysfunction. The measurement of extracellular pH levels can be used as the determination of glycolytic pathway utilization. Given the decrease in mitochondrial function seen on O2K respirometry, it will be useful to confirm that the glycolytic pathway is being used to generate ATP instead of the compromised cardiac mitochondria. Additionally, our study has some limitations. Some disease states predispose mitochondria to be more sensitive to disruption during preparation, and there are no bioenergetic living cell culture data with cardiomyocytes in a burn model. While our previous publication demonstrates some bioenergetic data [46,47] further study is needed to quantify tissue mitochondria.

## 5. Conclusions

In summary, we identified several significantly differentially expressed genes associated with metabolism following burn injury, such as the UCP1, Uqcrfs1, Atp12a, Ndufs4, and COX17 genes. These differences may be responsible for the pathogenesis of burn-induced cardiac mitochondrial dysfunction, while others may be compensatory mechanisms to protect the heart following a burn injury. The genes identified in this study constitute appropriate candidates for further study, particularly in testing the modulation of these pathways by pharmacotherapy.

## Figures and Tables

**Table 1:** Oligonucleotides used for the validation of the identified burn-inhibited mit gene expressions.

Gene	5′-Forward-3′	5′-Reverse-3′	Amplicon Size (bp)	Accession #
Atp5f1c	AGATGCATCGGTCATTGCCT	AGCCACGACGGTACTGAAAG	142	NM_053825.2
Atp5me	GTCACGGACAAAATGGTGCC	GTATGCCATGCCGAGGATCA	87	NM_080481.1
Atp5mg	GGCCAAGTTCATCCGTAACC	GAACCAGCTCAACCCTAGCA	116	NM_212516.2
Cox17	TGTGGACATCTCATCGAGGC	ATTCACAAAGTGGGCCACCA	82	NM_053540.2
Cox6c	CCACAGATGCGTGGTCTTCT	TCCTAGGGCCACAACGAATG	72	NM_019360.2
Cox 7a2	GAGTTCCGTTTCCGGTCTGG	CCTTCGTGAAGTGGTGCTGA	143	NM_022503.2
Ndufa8	GCCTGCCGTAAGCTAAGCAA	AGGGACCTGAGAGGTAACCG	105	NM_001047862.2
Ndufb3	CGCTAGTCCCGGAACGTTTA	TGACAGCAATGTGACCTCCC	132	NM_001106912.1
Ndufb7	AAGGTAGGGCAGAGTAGCCA	CTCCGGAAGACCGTAGTTCG	118	NM_001108442.1
Ndufb9	GAGCTGGGATCGGGAGGTTA	AAGTCGCCTTCCTTTCTGGC	96	NM_001127294.2
Ndufs4	TCCTGCTCGCAATAACATGC	CATGTTGGAGAGGGGATCGG	130	NM_001025146.1
Ndufs8	AGCCGCTGCACTTCAAGAT	ACTGCACTGCTATGAAGGCT	112	NM_001106322.2
Nqcrb	GGGATTAGGCAACCAGCACT	TCTGAAAGGCTGTGTCTCGG	116	NM_001127553.2

**Table 2:** Oligonucleotides used for the validation of the identified burn-stimulated mit gene expressions.

Gene	5′-Forward-3′	5′-Reverse-3′	Amplicon Size (bp)	Accession #
Atp12a	TCTTCGCCTTCACCACTCAG	GTTGAGGTGAGCCGGATAGG	149	NM_133517.2
Atp6v0a2	CTTCCGTAGCGAGAGCATGT	TTGAGGTCTCGGAACTGCAC	111	NM_053775.3
Atp6v1e2	GACCAAAGGCGGCTCCTG	ATGGCTGCTCTCAGGAATGG	132	NM_001108979.1
Atp6v1g3	TCCACCGAAGTTGCCTACAC	GCAGGTTCCTTCCTTTCCTCA	134	NM_001105991.1
Cox15	TGACATAGGCATTTTACTCTCCGA	TTCTGAGGTGAAGGGAGCCT	81	NM_001033699.4
Cox8c	TCCCAGCTGCGTATGTGATG	CAAGACGTTCAAACGGGCAC	148	NM_183055.1
Lhpp	CCTTCCGAGACAGTGGACG	ATCGGCAAAGCCTTGGGTAG	102	NM_001009706.1
Ndufa5	TCTACGTTCGATTGAGCGGG	CAATCCCACCAGGCCAGTAG	112	NM_012985.2
Slc25a10	CTCTAGACCTGCTCAAGGTGC	GAGGCACTCAGGCCATTGTA	124	NM_133418.1
Slc25a15	CTTGCTGTGTATCCGGTGGA	CTGACGTCATAGAGCCGTGA	149	NM_001047880.3
Ucp1	ATCCGGGCTTAAAGAGCGAG	CAGCCACCAGGGCTATTTGT	70	NM_012682.2
Ucp2	AGCAGTTCTACACCAAGGGC	TGGAAGCGGACCTTTACCAC	124	NM_019354.3
Ucp3	CGCCTGGAACAGAACAAAGC	TAACAGTGCAGGGTTCCGTC	77	NM_013167.2
Uqcrfs1	TGGCCATGTCGAAGATCGAG	TATGGCGCACAAACAGAGGT	96	NM_001008888.1
Ldha	ACCCTCTGGGGAATCCAGAA	ACACAACTGGACCAACTGGA	144	NM_017025.1

**Table 3:** Oligonucleotides used for the normalization of the identified burn-induced mit gene expressions.

Gene	5′-Forward-3′	5′-Reverse-3′	Amplicon Size (bp)	Accession #
β-actin	CTATGAGGGTTACGCGCTCC	ATGTCACGCACGATTTCCCT	141	NM_031144.3
B2m	CACTGAATTCACACCCACCG	TTACATGTCTCGGTCCCAGG	100	NM_012512.2
Gapdh	TTGTGCAGTGCCAGCCTC	GGTAACCAGGCGTCCGATAC	83	NM_017008.4
Hprt1	ACAGGCCAGACTTTGTTGGA	TGCCGCTGTCTTTTAGGCTT	149	NM 012583.2
Prlp0	TTGAACATCTCCCCCTTCTCCT	CCACATTGCGGACACCCTCTA	136	NM 022402.2

**Table 4:** Oligonucleotides used for the study of mtDNA copy number and gene expression.

Gene	5′-Forward-3′	5′-Reverse-3′	Amplicon Size (bp)	Accession #
ATP6	TAGGCTTCCGACACAAACTAAA	CTGCTAGTGCTATCGGTTGAATA	129	KF011917.1
ATP8	ATGCCACAACTAGACACAT	TTTGGGTGAGGGAGGTG	120	KF011917.1
COXI	GCCAGTATTAGCAGCAGGTATC	GGTGGCCGAAGAATCAGAATAG	125	KF011917.1
COXII	TCTCCCAGCTGTCATTCTTATTC	GCTTCAGTATCATTGGTGTCCTA	121	KF011917.1
COXIII	GCTGACCTCCAACAGGAATTA	CCTTCTATTAGGCTGTGATGGG	118	KF011917.1
Cyt B	CCTTCCTACCATTCCTGCATAC	TGGCCTCCGATTCATGTTAAG	118	KF011917.1
GAPDH	ACTCCCATTCTTCCACCTTTG	CCCTGTTGCTGTAGCCATATT	105	NM_017008.4
ND1	GGCTCCTTCTCCCTACAAATAC	AAGGGAGCTCGATTTGTTTCT	122	KF011917.1
ND2	CCCAACTATCACCACCATTCTC	TCGTGTTTGGGTCTGGTTAAG	79	KF011917.1
ND3	TTCTGCACGCCTTCCTTT	GGTTGTTTGAATCGCTCATGG	112	KF011917.1
ND4	GATGAGGCAACCAAACAGAAC	GTGTTGTGAGGGAGAGGATTAG	147	KF011917.1
ND4L	TCTCCTCTGCCTAGAAGGAATAA	TGGTAATTGGGATGGTTATGGAG	101	KF011917.1
ND5	GCCGCCACTATTATCTCCTTC	CTACTTCCTCCCACTCCATTTG	112	NM_133584.1
ND6	GGTGGGTTTGGATTGATTGTTAG	CCTCAGTAGCCATAGCAGTTG	148	NM_133584.1

## Abbreviations

ACTB	Beta-actin
B2M	Beta-2-microglobulin
C_T_	Cycle threshold
COX	Cytochrome c oxidase
GAPDH	Glyceraldehyde-3-phosphate dehydrogenase
HPRT1	Hypoxanthine phosphoribosyl transferase 1
mtDNA	Mitochondrial DNA
PCR	Polymerase chain reaction
RCR	Respiratory control ratio
RPLP0	Ribosomal protein: large, P0
SD	Standard deviation
TBSA	Total body surface body area
UCP	Uncoupling proteins

## References

[1] Fozzard H.A. (1961). Myocardial injury in burn shock. Ann. Surg..

[2] Howard T.S., Hermann D.G., McQuitty A.L., Woodson L.C., Kramer G.C., Herndon D.N., Ford P.M., Kinsky M.P. (2013). Burn-induced cardiac dysfunction increases length of stay in pediatric burn patients. J. Burn Care Res..

[3] Papp A., Uusaro A., Parviainen I., Hartikainen J., Ruokonen E. (2003). Myocardial function and haemodynamics in extensive burn trauma: Evaluation by clinical signs, invasive monitoring, echocardiography and cytokine concentrations. A prospective clinical study. Acta Anaesthesiol. Scand..

[4] Herndon D.N., Tompkins R.G. (2004). Support of the metabolic response to burn injury. Lancet.

[5] Guillory A.N., Clayton R.P., Herndon D.N., Finnerty C.C. (2016). Cardiovascular dysfunction following burn injury: What we have learned from rat and mouse models. Int. J. Mol. Sci..

[6] Bohanon F.J., Nunez Lopez O., Herndon D.N., Wang X., Bhattarai N., Ayadi A.E., Prasai A., Jay J.W., Rojas-Khalil Y., Toliver-Kinsky T.E. (2018). Burn Trauma Acutely Increases the Respiratory Capacity and Function of Liver Mitochondria. Shock.

[7] Padfield K.E., Astrakas L.G., Zhang Q., Gopalan S., Dai G., Mindrinos M.N., Tompkins R.G., Rahme L.G., Tzika A.A. (2005). Burn injury causes mitochondrial dysfunction in skeletal muscle. Proc. Natl. Acad. Sci. USA.

[8] Nakazawa H., Ikeda K., Shinozaki S., Kobayashi M., Ikegami Y., Fu M., Nakamura T., Yasuhara S., Yu Y.M., Martyn J.A.J. (2017). Burn-induced muscle metabolic derangements and mitochondrial dysfunction are associated with activation of HIF-1α and mTORC1: Role of protein farnesylation. Sci. Rep..

[9] Righi V., Constantinou C., Mintzopoulos D., Khan N., Mupparaju S.P., Rahme L.G., Swartz H.M., Szeto H.H., Tompkins R.G., Tzika A.A. (2013). Mitochondria-targeted antioxidant promotes recovery of skeletal muscle mitochondrial function after burn trauma assessed by in vivo 31P nuclear magnetic resonance and electron paramagnetic resonance spectroscopy. FASEB J..

[10] Brown D.A., O’Rourke B. (2010). Cardiac mitochondria and arrhythmias. Cardiovasc. Res..

[11] Bugger H., Abel E.D. (2010). Mitochondria in the diabetic heart. Cardiovasc. Res..

[12] Rosca M.G., Hoppel C.L. (2013). Mitochondrial dysfunction in heart failure. Heart Fail. Rev..

[13] Marín-García J., Akhmedov A., Rybin V., Moe G.W. (2013). Mitochondria and Their Role in Cardiovascular Disease.

[14] Zang Q., Maass D.L., White J., Horton J.W. (2007). Cardiac mitochondrial damage and loss of ROS defense after burn injury: The beneficial effects of antioxidant therapy. J. Appl. Physiol..

[15] Burch T.C., Rhim J.S., Nyalwidhe J.O. (2016). Mitochondria Biogenesis and Bioenergetics Gene Profiles in Isogenic Prostate Cells with Different Malignant Phenotypes. BioMed Res. Int..

[16] Tanaka T., Kobunai T., Yamamoto Y., Murono K., Otani K., Yasuda K., Nishikawa T., Kiyomatsu T., Kawai K., Hata K. (2017). Increased copy number variation of mtDNA in an array-based digital PCR assay predicts ulcerative colitis-associated colorectal cancer. In Vivo (Brooklyn).

[17] Li Z., Wang Q., Yu H., Zou K., Xi Y., Mi W., Ma Y. (2016). Screening of Key Genes in Severe Burn Injury at Different Stages via Analyzing Gene Expression Data. J. Burn Care Res..

[18] Liang W.Y., Tang L.X., Yang Z.C., Huang Y.S. (2002). Calcium induced the damage of myocardial mitochondrial respiratory function in the early stage after severe burns. Burns.

[19] Pesta D., Gnaiger E. (2012). High-resolution respirometry: OXPHOS protocols for human cells and permeabilized fibers from small biopsies of human muscle. Methods Mol. Biol..

[20] Laskowski K.R., Russell R.R. (2008). Uncoupling proteins in heart failure. Curr. Heart Fail. Rep..

[21] Kozak L.P., Anunciado-Koza R. (2008). UCP1: Its involvement and utility in obesity. Int. J. Obes..

[22] Liu S.-S. (1997). Generating, partitioning, targeting and functioning of superoxide in mitochondria. Biosci. Rep..

[23] Giordano F.J. (2005). Oxygen, oxidative stress, hypoxia, and heart failure. J. Clin. Investig..

[24] Givertz M.M., Colucci W.S. (1998). New targets for heart-failure therapy: Endothelin, inflammatory cytokines, and oxidative stress. Lancet.

[25] Liu Y., Fiskum G., Schubert D. (2002). Generation of reactive oxygen species by the mitochondrial electron transport chain. J. Neurochem..

[26] Kaminski M., Kiessling M., Suss D., Krammer P.H., Gulow K. (2007). Novel Role for Mitochondria: Protein Kinase C -Dependent Oxidative Signaling Organelles in Activation-Induced T-Cell Death. Mol. Cell. Biol..

[27] Zong N.C., Li H., Li H., Lam M.P.Y., Jimenez R.C., Kim C.S., Deng N., Kim A.K., Choi J.H., Zelaya I. (2013). Integration of cardiac proteome biology and medicine by a specialized knowledgebase. Circ. Res..

[28] Van Den Heuvel L., Ruitenbeek W., Smeets R., Gelman-Kohan Z., Elpeleg O., Loeffen J., Trijbels F., Mariman E., De Bruijn D., Smeitink J. (1998). Demonstration of a new pathogenic mutation in human complex I deficiency: A 5-bp duplication in the nuclear gene encoding the 18-kD (AQDQ) subunit. Am. J. Hum. Genet..

[29] Bugiani M., Invernizzi F., Alberio S., Briem E., Lamantea E., Carrara F., Moroni I., Farina L., Spada M., Donati M.A. (2004). Clinical and molecular findings in children with complex I deficiency. Biochim. Biophys. Acta Bioenerget..

[30] Sharma L., Lu J., Bai Y. (2009). Mitochondrial Respiratory Complex I: Structure, Function and Implication in Human Diseases. Curr. Med. Chem..

[31] Chang J., Jung H.J., Jeong S.H., Kim H.K., Han J., Kwon H.J. (2014). A mutation in the mitochondrial protein UQCRB promotes angiogenesis through the generation of mitochondrial reactive oxygen species. Biochem. Biophys. Res. Commun..

[32] Jung H.J., Cho M., Kim Y., Han G., Kwon H.J. (2014). Development of a novel class of mitochondrial ubiquinol-cytochrome c reductase binding protein (UQCRB) modulators as promising antiangiogenic leads. J. Med. Chem..

[33] Jung H.J., Kim K.H., Kim N.D., Han G., Kwon H.J. (2011). Identification of a novel small molecule targeting UQCRB of mitochondrial complex III and its anti-angiogenic activity. Bioorg. Med. Chem. Lett..

[34] Mansilla N., Racca S., Gras D.E., Gonzalez D.H., Welchen E. (2018). The complexity of mitochondrial complex iv: An update of cytochrome c oxidase biogenesis in plants. Int. J. Mol. Sci..

[35] Maxfield A.B., Heaton D.N., Winge D.R. (2004). Cox17 Is Functional When Tethered to the Mitochondrial Inner Membrane. J. Biol. Chem..

[36] Amaravadi R., Glerum D.M., Tzagoloff A. (1997). Isolation of a cDNA encoding the human homolog of COX17, a yeast gene essential for mitochondrial copper recruitment. Hum. Genet..

[37] Vitali M., Venturelli E., Galimberti D., Benerini Gatta L., Scarpini E., Finazzi D. (2009). Analysis of the genes coding for subunit 10 and 15 of cytochrome c oxidase in Alzheimer’s disease. J. Neural Transm..

[38] Capaldi R.A., Aggeler R., Turina P., Wilkens S. (1994). Coupling between catalytic sites and the proton channel in F1F0-type ATPases. Trends Biochem. Sci..

[39] Nijtmans L.G.J., Klement P., Houštěk J., van den Bogert C. (1995). Assembly of mitochondrial ATP synthase in cultured human cells: Implications for mitochondrial diseases. BBA Mol. Basis Dis..

[40] Zeviani M., Di Donato S. (2004). Mitochondrial disorders. Brain.

[41] Scudieri P., Musante I., Caci E., Venturini A., Morelli P., Walter C., Tosi D., Palleschi A., Martin-Vasallo P., Sermet-Gaudelus I. (2018). Increased expression of ATP12A proton pump in cystic fibrosis airways. JCI Insight.

[42] Crambert G. (2014). H-K-ATpase type 2: Relevance for renal physiology and beyond. Am. J. Physiol. Ren. Physiol..

[43] Knez J., Salvi E., Tikhonoff V., Stolarz-Skrzypek K., Ryabikov A., Thijs L., Braga D., Kloch-Badelek M., Malyutina S., Casiglia E. (2014). Left ventricular diastolic function associated with common genetic variation in ATP12A in a general population. BMC Med. Genet..

[44] Kinoshita K., Ashenagar M.S., Tabuchi M., Higashino H. (2011). Whole rat DNA array survey for candidate genes related to hypertension in kidneys from three spontaneously hypertensive rat substrains at two stages of age and with hypotensive induction caused by hydralazine hydrochloride. Exp. Ther. Med..

[45] Wen J.J., Cummins C.B., Szczesny B., Radhakrishnan R.S. (2020). Cardiac Dysfunction after Burn Injury: Role of the AMPK-SIRT1-PGCalpha-NFE2L2-ARE Pathway. J. Am. Coll. Surg..

[46] Wen J.J., Cummins C.B., Radhakrishnan R.S. (2020). Burn-Induced Cardiac Mitochondrial Dysfunction via Interruption of the PDE5A-cGMP-PKG Pathway. Int. J. Mol. Sci..

[47] Wen J.J., Cummins C., Radhakrishnan R.S. (2020). Sildenafil Recovers Burn-Induced Cardiomyopathy. Cells.

